# Deep neural network processing of DEER data

**DOI:** 10.1126/sciadv.aat5218

**Published:** 2018-08-24

**Authors:** Steven G. Worswick, James A. Spencer, Gunnar Jeschke, Ilya Kuprov

**Affiliations:** 1 School of Chemistry, University of Southampton, Highfield Campus, Southampton, SO17 1BJ, UK.; 2 Department of Chemistry and Applied Biosciences, Swiss Federal Institute of Technology in Zurich, Vladimir Prelog Weg 2, CH-8093 Zürich, Switzerland.

## Abstract

The established model-free methods for the processing of two-electron dipolar spectroscopy data [DEER (double electron-electron resonance), PELDOR (pulsed electron double resonance), DQ-EPR (double-quantum electron paramagnetic resonance), RIDME (relaxation-induced dipolar modulation enhancement), etc.] use regularized fitting. In this communication, we describe an attempt to process DEER data using artificial neural networks trained on large databases of simulated data. Accuracy and reliability of neural network outputs from real experimental data were found to be unexpectedly high. The networks are also able to reject exchange interactions and to return a measure of uncertainty in the resulting distance distributions. This paper describes the design of the training databases, discusses the training process, and rationalizes the observed performance. Neural networks produced in this work are incorporated as options into Spinach and DeerAnalysis packages.

## Teaser

Deep neural networks successfully solve Fredholm equations and extract molecular-scale distance distributions from EPR data.

## INTRODUCTION

Double electron-electron resonance (DEER), sometimes called pulsed electron double resonance (PELDOR), is a magnetic resonance experiment used to measure nanometer-scale distances between unpaired electrons in naturally paramagnetic or paramagnetically tagged systems (*1*, *2*). Extraction of distance information is possible because interelectron dipolar interaction energy is proportional to the inverse cube of the distance. Unlike scattering and diffraction methods, DEER does not require long-range order in the sample; it can be applied to a variety of systems that may not crystallize (*3*, *4*)—from molecular conductors (*5*) all the way to proteins and nucleic acids (*6*, *7*). Related methods, such as double-quantum electron paramagnetic resonance (DQ-EPR) (*8*, *9*) or relaxation-induced dipolar modulation enhancement (RIDME) (*10*, *11*), provide similar information. From a theoretical standpoint, DEER is quite straightforward: Its dipolar modulation signal factorizes into spin pair contributions, dipolar interactions with remote spins are the only significant signal decay mechanism, and the broadening caused by that decay can be deconvolved because the decay function is available from the unmodulated background (*12*).

DEER spectroscopy involves recording a dipolar modulation signal between two unpaired electrons and running regularized fitting to extract the distance distribution (*13*, *14*). The procedure works well in spin-½ systems (*15*), but significant complications arise when (i) more than two electron spins are present (*16*, *17*), (ii) the total spin of any paramagnetic center exceeds ½ (*18*, *19*), (iii) large interaction tensor anisotropies generate orientation selection effects (*20*, *21*), (iv) the system has microsecond-scale internal dynamics, and (v) the system has significant interelectron exchange coupling (*22*, *23*). Some of these matters are exceedingly hard to resolve or work around. It is also becoming clear that ab initio modeling and fitting of every possible complication are out of the question.

In this communication, we report an attempt to train deep neural networks to convert DEER signals into spin label distance distributions. DEER data processing is well suited for the application of supervised learning techniques because it is a simple “vector-in, vector-out” regression problem (*24*). We used a large training database of synthetic DEER traces computed using Spinach (*25*) from randomly generated realistic distance distributions with a variable baseline and a variable amount of noise. The objective is to train networks that would recognize and work around all of the issues mentioned above; here, we address complicated distance distributions, exchange coupling, baseline distortions, and noise.

We found that neural networks successfully process previously unseen experimental data in the presence of exchange coupling, as well as realistic amounts of noise and baseline signal. They are also able to provide a measure of confidence in the output. Once the training process is finished, the networks have no adjustable parameters. In cases where a stable or a regularizable solution exists in principle, we expect that neural networks should eventually be able to solve most of the above problems (i) to (v) when they are trained on a database of sufficient size and scope.

### DEER data processing—State of the art

For an isolated electron pair, at a distance *r* with isotropic magnetogyric ratios γ_1_ and γ_2_, the echo modulation signal has the following form (see the Supplementary Materials for detailed derivations)

$$s(r,\theta ,t)=\;\text{cos}[(D[1-3{\text{cos}}^{2}(\theta )]+J)t],\;D=\frac{\mu_{0}}{4\pi }\frac{\gamma_{1}\gamma_{2}\hbar }{r^{3}}$$ (1)

where *J* is the exchange coupling (nuclear magnetic resonance convention) and θ is the angle between the interelectron direction and the magnet field. A typical experimental system is a frozen glass with all orientations equally likely. Integrating Eq. 1 over all angles produces a function known as the DEER kernel

$$\gamma (r,t)=\sqrt{\frac{\pi }{6Dt}}[\text{cos}[(D+J)t]\text{FrC}[\sqrt{\frac{6Dt}{\pi }}]+\;\text{sin}[(D+J)t]\text{FrS}[\sqrt{\frac{6Dt}{\pi }}]]$$ (2)

in which FrC and FrS are Fresnel’s cosine and sine functions. For an ensemble of isolated spin-½ pairs, the experimentally observed DEER trace is an integral of the kernel over the distance distribution

$$d(t)=\int_{0}^{\infty }p(r)\gamma (r,t)dr$$ (3)

Even in this ideal case, the relationship between the distance distribution *p*(*r*) and the experimental signal *d*(*t*) is not straightforward: It is an integral whose inversion is an ill-posed problem.

The most popular procedure for extracting distance distributions from DEER traces of real systems (*13*–*15*) rests on a number of significant assumptions. The primary one is the dilute spin pair approximation—it is assumed that the dipolar evolution function *d*(*t*) may be modeled as a linear combination of DEER traces of systems involving point electrons at specific distances (*4*). Equation 2 is strictly valid only for spin-½ paramagnetic centers. For higher spin quantum numbers, this model only applies in the absence of level mixing and when overtone transitions during the pump pulse can be neglected. Exchange coupling is also commonly ignored, which often, but not always, provides a good approximation at distances longer than 15 Å (*22*).

The next assumption deals with nonideal pulses and the inevitable presence of external interactions. For dilute spin pairs, the experimental DEER signal *v*_exp_(*t*) can be approximated as

$$v_{\text{exp}}(t)=[1-\lambda +\lambda d(t)]b(t)+n(t)$$ (4)

where *n*(*t*) is the instrument noise, λ is the spin-flip probability under the action of the pump pulse (*12*, *26*), and *b*(*t*) is the intermolecular background function—usually a stretched exponential

$$b(t)=\;\text{exp}[-{\left(kt\right)}^{N/3}]$$ (5)

that corresponds to a homogeneous distribution of distant spins in a space with dimension *N* (*27*). Equation 5 is also a good approximation for a homogeneous distribution in three dimensions with some excluded volume around the observer molecule (*28*). Along with relaxation, the background function limits the observation time and puts an upper limit on the distances that can be measured (*12*, *29*).

Even after *b*(*t*) and λ are obtained by fitting, the mapping back from *d*(*t*) into *p*(*r*) is still unstable—an infinitesimally small variation in *d*(*t*) can cause a finite variation in *p*(*r*). Tikhonov regularization is therefore commonly used, in which the ambiguity is removed by requiring the second derivative of the solution to have the minimum norm (*13*, *14*, *30*). This requirement incorporates the physical wisdom that the solution must be smooth and sparse. The combined fitting error functional is

$$\Omega [p(r)]={\left\|d_{\text{exp}}(t)-\int_{0}^{\infty }p(r)\gamma (r,t)dr\right\|}^{2}+\alpha {\left\|\frac{d^{2}}{{dr}^{2}}p(r)\right\|}^{2}$$ (6)

where α is the regularization parameter, chosen using the L-curve method (*14*, *31*). Other regularization methods have also been tried and generally found to be successful (*32*, *33*).

Regularization makes the problem tractable, but some distortions are inevitable: Narrow features are broadened, and broad features are artificially split. The error minimization runs within a reasonable length of time when an analytical expression for γ(*r*, *t*) is available. When that is not the case (for example, in high-spin systems), the process becomes impractically slow, even on the latest computing hardware and software (*19*).

When the experimental DEER trace and the associated distance distribution are discretized on finite grids, Eq. 6 acquires a matrix-vector form

$$\Omega [p]={\left\|d_{\text{exp}}-\Gamma p\right\|}^{2}+\alpha {\left\|D^{2}p\right\|}^{2}$$ (7)

where **Γ** is the matrix form of the DEER kernel integral and **D** is a derivative matrix—for example, a finite difference one. At this point, we have a standard Tikhonov problem with a non-negativity constraint that is also encountered elsewhere in magnetic resonance (*34*, *35*). Bayesian methods exist for uncertainty estimation (*36*), and the widely used DeerAnalysis package includes a validation tool (*12*).

**Fig. 1:**
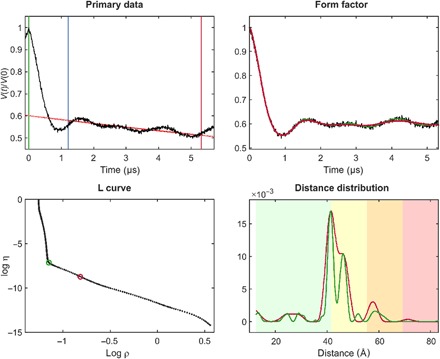
Standard Tikhonov regularization processing, illustrated using site pair V96C/I143C in the lumenal loop of a double mutant of LHCII, with iodoacetamido-PROXYL spin labels attached to the indicated cysteines (*64*). For the primary data (top left), the zero time (green vertical line) is determined using moment analysis in the vicinity of the intensity maximum. The optimal starting time for background fitting (blue vertical line) is determined by minimizing probability density at the maximum distance. Data have been cut by 400 ns at the end (red vertical line) to minimize the influence of the artifact arising from overlapping pump and observe pulse excitation bands. The stretched exponential background fit is shown as a solid red line (where fitted) and as a dotted red line (where extrapolated). The background-corrected data (form factor, black) are shown in the top right panel together with fits using the regularization parameter corresponding to the origin distance criterion (red) and maximum curvature criterion (green). These two choices are also indicated in the L-curve (bottom left). The bottom right panel shows distance distributions computed with these two regularization parameters in matching color. Pastel background shading indicates distance ranges where the shape of the distribution is expected to be reliable (green), where mean distances and widths are expected to be reliable (yellow), where only mean distances are expected to be reliable (orange), and where data should not be interpreted (red). These ranges are derived from the duration of the primary data (*7*).

The regularized fitting method, as illustrated in Fig. 1, works very well for simple spin-½ systems (*37*, *38*). Limited workarounds are available for situations when the core assumptions behind Eqs. 1 to 5 do not hold. For multispin systems, data closer to the isolated spin pair approximation can be obtained by intentionally reducing modulation depth (*16*), by power scaling (*17*), or by sparse spin labeling (*39*). For Gd(III) with spin 7/2, researchers have demonstrated that distortions caused by level mixing can be reduced by large frequency offsets between pump and observe pulses (*40*) or by RIDME (*41*). The latter technique introduces overtones of the dipolar frequency (*42*) that require a modified DEER kernel with overtone coefficients that must be calibrated (*43*). Deviations from the isotropic distribution of the spin-spin vector by orientation selection can be partially averaged by varying the magnetic field at constant pump and observe frequencies (*37*, *44*). In some site-directed spin labeling applications, an experimental estimate of the background can be obtained by measuring singly labeled constructs (*15*). Significant progress was also recently made with Mellin transform techniques (*45*) that are likely to improve further once the non-negativity constraint is introduced.

### Connection to neural networks

The previous section describes a process that alternates matrix-vector operations with nonlinear constraints—a good match to the algebraic structure of a feedforward neural network (*46*)

$$x_{n}=g_{n}(W_{n}x_{n-1}+y_{n})$$ (8)

where the *n*th neuron layer accepts an input vector **x**_*n* − 1_, multiplies it by a weight matrix **W**_*n*_, adds a bias vector **y**_*n*_, and passes the result through a nonlinear transfer function *g*_*n*_. This similarity is not strictly necessary—McCulloch and Pitts (*47*) showed that neural networks can compute any arithmetical or logical function. Multilayer feedforward networks are known to be universal approximators (*46*), but the present case is particularly appealing because the required network is likely to be quite small.

DEER signals contain true dipolar oscillation, a background signal, and a noise track that are statistically independent. The task of reconstructing a distance distribution can therefore be broken down into performing, in the least-squares sense, the following operations

$$\begin{array}{cc} \left[1-\lambda +\lambda d_{i}]⊙b_{j}=N^{-1}([1-\lambda +\lambda d_{i}]⊙b_{j}+n_{k}\right) & \forall i,j,k\\ d_{i}=B^{-1}([1-\lambda +\lambda d_{i}]⊙b_{j}) & \forall i,j\\ p_{i}=\Gamma^{-1}d_{i} & \forall i \end{array}$$ (9)

where ⊙ denotes element-by-element multiplication, **N**^− 1^ may be called “denoising,” **B**^− 1^ may be called “background rejection,” and **Γ**^− 1^ may be called “interpretation.” All three operations are not necessarily described by matrices, are ill-posed, and only exist in the least-squares sense over an infinitely large number of instances of the true DEER signal **v**_*i*_, the background signal **b**_*j*_, and the noise signal **n**_*k*_.

All three operations are linear with respect to the dipolar modulation signal and are nonlinear with respect to the background and the noise. They map well into Eq. 8 and the neural network training process. Large databases of {**p**_*i*_, **d**_*i*_, **b**_*j*_, **n**_*k*_} can be generated using Spinach (*25*), and the networks performing **N**^−1^, **B**^−1^, and Γ^−1^ can be obtained using backpropagation training (*48*, *49*). These networks are called mapping networks; they are extensively researched (*46*, *47*, *50*).

At a more general level, neural network “surrogate” solutions to Fredholm equations are well researched in their own right (*51*), with rigorous accuracy bounds available (*52*, *53*). In 2013, Jafarian and Nia (*54*) proposed a two-layer feedback network built around a Taylor expansion of the solution; Effati and Buzhabadi (*55*) published a feedforward network proposition. Both groups considered a generic Fredholm equation without any specific physical model or context. At that time, neither group had the computing power to train a network of sufficient width and depth to perform the tasks encountered in this work. However, both groups observed that, for such problems as they could handle, neural networks provided very accurate solutions (*54*, *55*). Promising neural network results also exist for two-dimensional (2D) integral equations (*56*, *57*), meaning that processing triple electron resonance spectroscopy (*58*) data with neural networks may also be possible.

## MATERIALS AND METHODS

### Training database generation

**Fig. 2:**
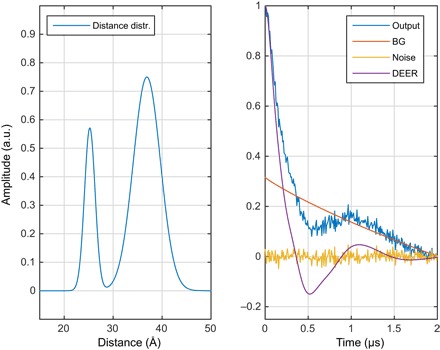
One of the millions of synthetic DEER data sets, generated using Spinach (*25*) and used for neural network training in this work. (**Left**) Randomly generated distance distribution. (**Right**) The corresponding DEER form factor (purple), a randomly generated noise track (yellow), a randomly generated intermolecular background signal (red, marked BG), and the resulting “experimental” DEER signal (blue). a.u., arbitrary units.

Neural network training requires a library of inputs and their corresponding outputs covering a range that is representative of all possibilities (*48*, *49*, *59*). Real distance distributions between spin labels are rarely known exactly and, therefore, collating experimental data is not an option. Fortunately, high-accuracy simulations, taking into account most of the relevant effects, have recently become possible (*19*, *25*, *60*). They can be time-consuming (*19*) but only need to be run once to generate multiple simulated DEER traces with different artificial noise and background functions. These traces are then stored in a database alongside the “true” distance distributions they were generated from. An example is shown in Fig. 2.

The size and shape of the training database are entirely at the trainer’s discretion—a wide variety of spin systems, parameter ranges, secondary interactions, and instrumental artifacts may be included. This exploratory work uses the DEER kernel for a pair of spin-½ particles, but the DEER simulation module in Spinach is not restricted in any way (*60*)—training data sets may be generated for any realistic combinations of spins, interactions, and pulse frequencies. The following parameters are relevant:

(1) Minimum and maximum distances in the distribution. Because the dipolar modulation frequency is a cubic function of distance, there is a scaling relationship between the distance range and the signal duration

$$\frac{t_{A}}{r_{A}^{3}}=\frac{t_{B}}{r_{B}^{3}}$$ (10)

The salient parameter here is the “dynamic range”—the ratio of the longest distance and the shortest. Training signals must be long enough and discretized well enough to reproduce all the frequencies present.

(2) Functions used to represent distance peaks and their number. A random number of skew normal distribution functions (*61*) with random positions within the distance interval and random full widths at half magnitude were used in this work

$$p(x)=\frac{2}{\sigma \sqrt{2\pi }}e^{-\frac{{\left(x-x_{0}\right)}^{2}}{{2\sigma }^{2}}}\int_{-\infty }^{\alpha (\frac{x-x_{0}}{\sigma })}e^{-\frac{t^{2}}{2}}dt$$ (11)

where σ is the SD of the underlying normal distribution, *x*_0_ is the location of its peak, and α is the shape parameter regulating the extent of the skew. Distance distributions were integrated with the DEER kernel in Eq. 2 to obtain DEER form factors. We found that generating distance distributions with up to three peaks was sufficient to ensure that the networks could generalize to an arbitrary number of distances (see the “Measures of uncertainty” section).

(3) Noise parameters and modulation depth. Because DEER traces were recorded in the indirect dimension of a pseudo-2D experiment, the noise was not expected to be colored—this was confirmed by experiments (*36*). We used Gaussian white noise with the SD chosen randomly between zero and a user-specified fraction of the modulation depth, which was also chosen randomly from within the user-specified ranges.

(4) Background function model and its parameters. We used Eq. 5 with the dimensionality parameter selected randomly from the user-specified range.

(5) Discretization grids in the time and the distance domains. The point count must be above the Nyquist condition for all frequencies expected within the chosen ranges of other parameters. The number of discretization points dictates the dimension of the transfer matrices and bias vectors in Eq. 8, which, in turn, determine the minimum training set size.

(6) Training set size. A fully connected neural network with *n* layers of width *k* has *n*(*k*^2^ + *k*) parameters. Each of the “experimental” DEER traces is *k* points long, meaning that *n*(*k* + 1) is the absolute minimum number of DEER traces in the training set. At least 100 times that amount is in practice necessary to generate high-quality networks.

The parameter ranges entering the training data set are crucial for the success of the resulting network ensemble—the training data set must be representative of the range of distances, peak widths, noise amplitudes, and other attributes of the data sets being processed. The parameters entering the current DEERNet training database generation process are listed in Table 1.

**Table 1:** Training database generation parameters used in this work. Where a maximum value and a minimum value are given, the parameter is selected randomly within the interval indicated for each new entry in the database. Ranges in the suggested values indicate recommended intervals for the corresponding parameter.

**Parameter**	**Suggested values**
Minimum distance in the distribution (Å)	10–15
Maximum distance in the distribution (Å)	50–80
DEER trace length (μs)	2–5
Minimum number of distance peaks	1–2
Maximum number of distance peaks	2–3
Data vector size	256–1024
RMS noise, fraction of the modulation depth	0.05–0.10
Minimum exchange coupling (MHz)	−5.0
Maximum exchange coupling (MHz)	+5.0
Minimum background dimensionality	2
Maximum background dimensionality	3.5
Minimum full width at half magnitude for distance peaks, fraction of the distance	0.05–0.10
Maximum full width at half magnitude for distance peaks, fraction of the distance	0.20–0.50
Maximum shape parameter (Eq. 11)	+3.0
Minimum shape parameter (Eq. 11)	−3.0
Minimum modulation depth	0.05–0.10
Maximum modulation depth	0.50–0.60
Minimum background decay rate (MHz)	0.0
Maximum background decay rate (MHz)	0.5

Reliable neural network training requires signals in the database to be consistently scaled and to fall within the dynamic range of the transfer functions. The peak amplitude of each distance distribution was therefore brought by uniform scaling to 0.75, and all DEER traces were uniformly scaled and shifted so as to have the first point equal to 1 and the last point equal to 0.

The training process requires vast computing resources, but using the trained networks does not. For the networks and databases described in this communication, the training process for a 100-network ensemble takes about a week on a pair of NVidia Tesla K40 cards. Once the training process is finished, the networks can be used without difficulty on any computer strong enough to run MATLAB.

### Network topology and the training process

**Fig. 3:**
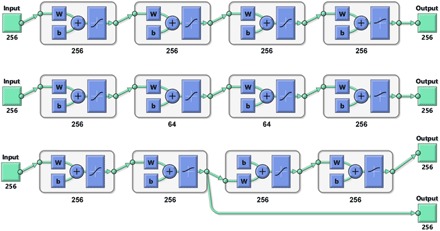
Schematic diagrams (produced by MATLAB) of the three types of neural network topologies explored in this work, using four-layer networks as an example. W block indicates multiplication by the weight matrix and b block indicates the addition of a bias vector. (**Top**) Fully connected full-width network. (**Middle**) Fully connected network with choke points. (**Bottom**) Functionally separated network with some layers explicitly dedicated to background rejection and others to interpretation—during the training process, the first output is the DEER form factor, and the second output is the distance probability density function.

Three simple types of feedforward network topologies explored in this work are shown in Fig. 3. Basic fixed width feedforward networks (top diagram) do, in practice, suffice, but we have also explored variable width networks (middle diagram) and networks based on the stage separation discussed around Eq. 9. Specifically, it makes physical sense to separate the form factor extraction stage from the DEER signal interpretation stage (Fig. 3, bottom diagram).

The most common transfer functions in Eq. 8 are sigmoidal, mapping [− ∞, ∞] into [− 1, 1]. However, distance distribution is a non-negative function, and we observed that including this fact at the network level improves performance. Using the strictly positive logistic sigmoid function

$$g(x)=\frac{1}{1+e^{-x}}$$ (12)

at the last layer instead of the hyperbolic tangent function used by the inner layers

$$g(x)=\frac{e^{x}-e^{-x}}{e^{x}+e^{-x}}$$ (13)

decreases both the final error and the training time (table S1).

The training of all neural networks was carried out on NVidia Tesla K20 and K40 coprocessor cards using MATLAB R2018a Neural Network Toolbox and Distributed Computing Toolbox. Resilient backpropagation (*49*) and scaled conjugate gradient (*48*) error minimization methods were used with the least-squares error metric. Training databases were partitioned into a 70% training set (with respect to which the minimization was carried out), a 15% validation set (that was monitored to prevent overfitting), and a 15% testing set with respect to which the performance figures were compiled; this is in line with standard practice.

### Uniform feedforward networks

**Fig. 4:**
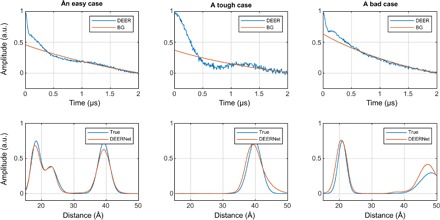
Distance distribution recovery performance illustration for a five-layer feedforward neural network, fully connected, with 256 neurons per layer. All inner layers have hyperbolic tangent transfer functions; the last layer has the strictly positive logistic sigmoid transfer function.

**Fig. 5:**
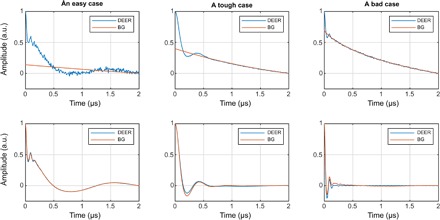
DEER form factor recovery performance illustration for a six-layer feedforward neural network, fully connected, with 256 neurons per layer. All layers have hyperbolic tangent transfer functions.

The simplest strategy for training a generic “vector-in, vector-out” neural network is to set up a number of fully connected layers of the same size as the input vector, resulting in the topology shown in the top diagram of Fig. 3. The performance metrics for a family of such networks are given in Table 2 and illustrated graphically in Figs. 4 and 5. The “relative error” metric is defined as the 2-norm of the difference between the network output and the true answer divided by the 2-norm of the true answer.

**Table 2:** Performance statistics for a family of feedforward networks set up as a simple sequence of fully connected layers of the same width as the input vector. A schematic of the network topology is given in the top diagram of Fig. 3.

**Task**	**Network**	**Mean relative error**	**Relative error SD**	Iteration time*, Tesla K40 (s)
Distance distribution recovery	In-(256)_2_-Out	0.090	0.231	0.32
	In-(256)_3_-Out	0.077	0.208	0.44
	In-(256)_4_-Out	0.070	0.195	0.74
	In-(256)_5_-Out	0.069	0.194	0.99
	In-(256)_6_-Out	0.069	0.192	1.19
Form factor recovery	In-(256)_2_-Out	0.0065	0.0143	0.31
	In-(256)_3_-Out	0.0042	0.0094	0.51
	In-(256)_4_-Out	0.0037	0.0084	0.75
	In-(256)_5_-Out	0.0034	0.0080	0.98
	In-(256)_6_-Out	0.0034	0.0080	1.18

*Using a database with 100,000 DEER traces generated as described under “Training database generation” section.

It is clear from the performance statistics that, for a single neural network, the average norm of the deviation drops below 10% of the total signal norm and stops improving once the network is five to six layers deep. Training iteration time depends linearly on the depth of the network.

The data for the visual performance illustrations (Figs. 4 and 5) were selected from the training database in the following way: the “easy case” was sampled from the relative error histogram region located between 0 and 1 SD; the “tough” case was sampled from the region between 1 and 2 SDs; the “bad case” was sampled from 100 worst fits in the entire 100,000-trace training database. Performance illustrations for the rest of the networks reported in Table 2 are given in figs. S1 to S3. Given that the bad cases are the worst 0.1% of the training data set, the performance is rather impressive. Similar sequential improvements are observed for the networks tasked with the recovery of the DEER form factor (Fig. 5).

For the vast majority of DEER traces in the training database, the recovery of the form factor is close to perfect. Performance illustrations for the rest of the form factor recovery networks reported in Table 2 are given in figs. S4 to S6.

### Feedforward networks with choke points

Excellent as the performance of the neural networks in Table 2 and Fig. 4 may appear, deeper inspection still indicates that having 256 neurons in the inner layers may not be necessary, and this dimension can potentially be reduced. This is most obvious from the analysis of singular value decompositions (SVDs) of the weight matrices in Eq. 8. The general form of the SVD of a matrix **W** is

$$W=\sum_{k}\sigma_{k}|u_{k}\rangle \langle v_{k}|$$ (14)

where the right singular vectors 〈*v*_*k*_| may be viewed as a library of distinct input signals, the left singular vectors |*u*_*k*_〉 may be viewed as the library of distinct output signals, and the singular values *σ*_*k*_ may be viewed as the amplification coefficients applied when an input is mapped into an output. If some singular values are zero, then the corresponding pathways are unimportant and may be dropped. Mathematically, this means that the rank of the matrix is smaller than its dimension.

**Fig. 6:**
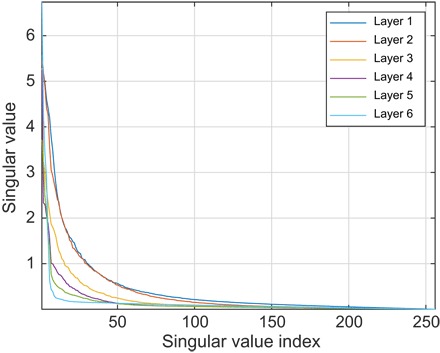
Singular values of the weight matrices in a six-layer feedforward neural network, fully connected, with 256 neurons per layer, and trained as described in the main text. All inner layers have hyperbolic tangent transfer functions; the last layer has the strictly positive logistic sigmoid transfer function.

Singular values of all transfer matrices in a six-layer distance distribution recovery network are plotted in Fig. 6. It is clear that none of the weight matrices are full rank, and the matrices occurring later in the network have fewer large-amplitude singular values. This suggests that intermediate layers could require fewer than 256 neurons. Because the corresponding singular values are small or zero, reducing the number of neurons in intermediate layers is not expected to affect accuracy. However, the reduction in the training time could be considerable: A fully connected *N*-neuron layer has *N*^2^ + *N* adjustable parameters, and so the benefit of going down from 256 neurons to 64 or fewer is significant.

This is explored in detail in Table 3. Although the intuition provided by Eq. 14 and Fig. 6 suggests that reducing the number of neurons in the intermediate layers might be a good idea, this is not corroborated by the practical performance figures. Any reduction in the dimension of intermediate layers results in performance degradation. The position of the choke point (table S2) does not appear to have any influence on the performance.

**Table 3:** Performance statistics for a family of feedforward networks set up as a simple sequence of fully connected layers with a choke point in the middle. A schematic of the network topology is given in the middle diagram of Fig. 3.

**Network**	**Mean relative error**	**Relative error SD**	Iteration time*, Tesla K40 (s)
In-256-***32***-256-Out	0.095	0.230	0.25
In-256-***64***-256-Out	0.086	0.217	0.29
In-256-***128***-256-Out	0.084	0.217	0.39
In-256-***256***-256-Out	0.077	0.208	0.51
In-(256)_2_-***32***-(256)_2_-Out	0.090	0.210	0.61
In-(256)_2_-***64***-(256)_2_-Out	0.074	0.201	0.65
In-(256)_2_-***128***-(256)_2_-Out	0.073	0.200	0.83
In-(256)_2_-***256***-(256)_2_-Out	0.069	0.194	0.99

*Using a database with 100,000 DEER traces generated as described under “Training database generation” section.

Another architectural observation is that bias vectors do not appear to be necessary in Eq. 8—networks trained without bias vectors have identical performance (table S2). An examination of the optimal bias vectors does not yield any interpretable patterns. This is likely because the input and the output data are already well scaled (see “Training database generation” section) and fit into the dynamic window of the transfer functions without the need for any shifts. Still, the variational freedom afforded by the bias vectors appears to accelerate the training process, and we have kept them for that reason.

### Structured networks

Table 2 indicates that plain feedforward networks with more than six layers do not produce any further improvements in the performance. If those improvements are even possible, then more sophisticated topologies must be used. One possibility is shown in the bottom diagram of Fig. 3—the first group of layers was trained against the form factor and therefore eliminated noise and background. That form factor was then fed into the second group of layers, making the probability density extraction easier for those layers. In principle, structured networks may be assembled from pretrained pieces. In the case of the bottom diagram of Fig. 3, the pieces would come from one of the form factor extraction networks in Table 1 and a separate network trained to interpret background-free form factors. Performance figures for networks of this type are given in Table 4.

**Table 4:** Performance statistics for a family of tailored networks composed of a group of form factor extraction layers that form the input of the interpretation layers. A schematic of the network topology is given in the bottom diagram of Fig. 3. FF, form factor; Int, interpretation.

**Network topology**	**Interpretation**		**Form factor extraction**	
	**Mean relative error**	**Relative error SD**	**Mean relative error**	**Relative error SD**
In-FF[(256)_1_]-Int[(256)_1_]-Out	0.276	0.467	0.355	0.383
In-FF[(256)_2_]-Int[(256)_2_]-Out	0.103	0.236	0.040	0.083
In-FF[(256)_3_]-Int[(256)_3_]-Out	0.094	0.225	0.021	0.046
In-FF[(256)_4_]-Int[(256)_4_]-Out	0.087	0.216	0.015	0.028
In-FF[(256)_5_]-Int[(256)_5_]-Out	0.081	0.196	0.012	0.023
In-FF[(256)_6_]-Int[(256)_6_]-Out	0.080	0.192	0.012	0.022

Unfortunately, it does not appear that tailoring carries any advantages relative to the data reported for the simple feedforward networks in Table 2. Training a 12-layer network against two sets of outputs is also exceedingly expensive. We therefore used uniform feedforward networks (Fig. 3, top) for all production calculations discussed below. The networks were trained on a data set where raw experimental data without any preprocessing go in, and the distance distribution is expected at the output.

Still, the networks evaluated in Table 4 could potentially be beneficial as a safety catch: Humans can easily recognize incorrect form factors visually and thus detect cases of neural networks failing, for example, if they encounter a situation not covered by the training set.

### Measures of uncertainty

When applied correctly, the standard Tikhonov regularized DEER data analysis (*12*–*14*) produces clear results and easily interpretable distance distributions. However, when applied naively to corrupted or featureless data sets, it can result in overinterpretation of the data (*12*, *36*, *38*). In particular, less experienced practitioners may have difficulty distinguishing genuine distance peaks from artifacts (*62*). Feedback from the EPR community has led to the concept of a validation tool that would be able to identify corrupted or featureless DEER traces. These tools exist within the Tikhonov framework (*12*, *36*), although they can be computationally demanding. A similar tool is therefore required for neural networks.

A {“good”, “bad”} classification network would be the obvious solution, but the amount of experimental DEER data in the world is rather small—polling the community for examples of bad DEER traces is unlikely to return a data set of sufficient size. We therefore decided to pursue another common alternative: to train an ensemble of neural networks using different synthetic databases and to use the variation in their outputs as a measure of uncertainty in the distance distribution (*63*). Such a measure is useful in any case, and a large variation would indicate uninterpretable input data.

To investigate the performance of this approach in estimating distance distribution uncertainties and detecting corrupted data, we trained 100 five-layer networks on different databases (generated as described under “Training database generation” section) and evaluated their performance against a previously unseen database.

**Fig. 7:**
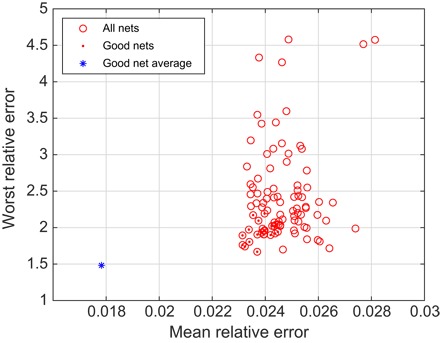
Performance of an ensemble of 100 five-layer neural networks on a previously unseen database. Each of the networks was started from a different random initial guess and trained in a different randomly generated database. Red dots indicate the good networks that are better than the median on both the mean relative error and the worst relative error. The blue asterisk is the performance of the average output of the good networks.

**Fig. 8:**
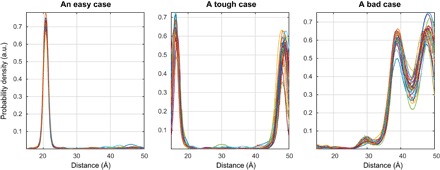
Network ensemble performance illustration. Easy (**left**), tough (**middle**), and worst-case (**right**) agreement on the training set data. The variation in the outputs of different neural networks within the ensemble is a measure of the uncertainty in their output (*63*) when the training databases are comprehensive.

The results are shown in Fig. 7. The relative error metric is the ratio of the 2-norm of the difference between the output and the true answer divided by the 2-norm of the true answer. The “worst relative error” refers to the worst-case performance in the entire database. Performance metrics for all networks in the ensemble are plotted as red circles. The networks that scored better than the median on both characteristics are labeled good and additionally marked with a dot. The performance of the arithmetical mean of the outputs of good networks is shown as a blue asterisk. The SD of the mean across the good network ensemble is a measure of uncertainty in the output (Fig. 8).

In practice, the mean output signal and the SD are computed for each point and plotted in the form of 95% confidence bounds, as shown in the figures presented in the next section. A more detailed investigation of the effect of the noise in the input data on the reconstruction quality and the confidence intervals is given in section S5.

**Fig. 9:**
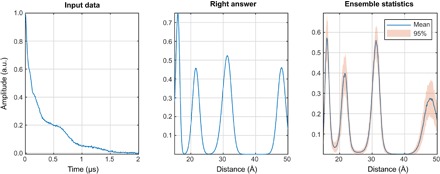
A demonstration that deep neural networks learn to be Fredholm solvers rather than model fitters. Presenting a data set with four distances to networks trained on the database with at most three distances yields the right answer with high confidence. All networks in the ensemble return four peaks.

An important practical test of correctness, intended to distinguish a neural network that merely fits a few Gaussians to the data set from a network that is a Fredholm solver, would be to present a DEER trace with four distances to a network that was trained on a database with at most three. A network that has learned to be a Fredholm solver in the sense discussed in (*51*, *52*, *54*, *55*, *57*) should still return the right answer. As Fig. 9 illustrates, our networks pass that test.

## RESULTS AND DISCUSSION

This section contains a demonstration of the practical performance of neural network ensembles for distance distribution reconstruction and uncertainty analysis. The results from the best current Tikhonov method implementation (*15*) are provided as a reference.

### Test case library

DEER is used most widely in structural biology on doubly spin-labeled proteins, nucleic acids, and their complexes. In some cases, distance distributions are narrow and give rise to time-domain data with several observable oscillations. As an example, we use DEER data for site pair 96/143 in the monomeric plant light-harvesting complex II (LHCII; sample I) (*64*). When intrinsically disordered domains are present, distance distributions can be very broad. This applies to site pair 3/34 in LHCII (sample II) (*64*). Even narrower and broader distributions are found in polymer science. We encountered the smallest width-to-distance ratio in a short oligo-phenyleneethynylene end-labeled with a rigid nitroxide label (sample III) (*37*). One of the broadest distributions for which we have high-quality DEER data was observed in a [2]catenane spin-labeled on both of the intertwined macrocycles (sample IV) (*65*). As an example, where a narrow and a broad distance distribution peak are simultaneously present, we use decorated gold nanoparticles (sample V) (*66*). As a typical example for the distributions encountered in large rigid organic molecules, we use a doubly labeled phenyleneethynylene molecule (sample VI) (*16*).

### Experimental data preprocessing

We preprocessed all primary data in DeerAnalysis (*12*). We accepted the zero time of the dipolar oscillation and signal phase determined automatically by DeerAnalysis. We cut off the last 400 ns of each trace to remove the “2 + 1” end artifact that arises from excitation band overlap of pump and observe pulses (*7*). For sample III, a part of the end artifact was still visible, and the last 800 ns had to be cut off. These data were supplied to DEERNet, which expects a column vector containing the time axis (from 0 to *t*_max_) in microseconds and a column vector of the corresponding DEER signal amplitudes. Internally, the signal is shifted and scaled to match the dynamic range of the network, and downsampled with a matched quadratic Savitzky-Golay filter to make the number of points equal to the number of neurons in the input layer. The trace length *t*_max_ is used in Eq. 10 to determine the distance axis.

**Fig. 10:**
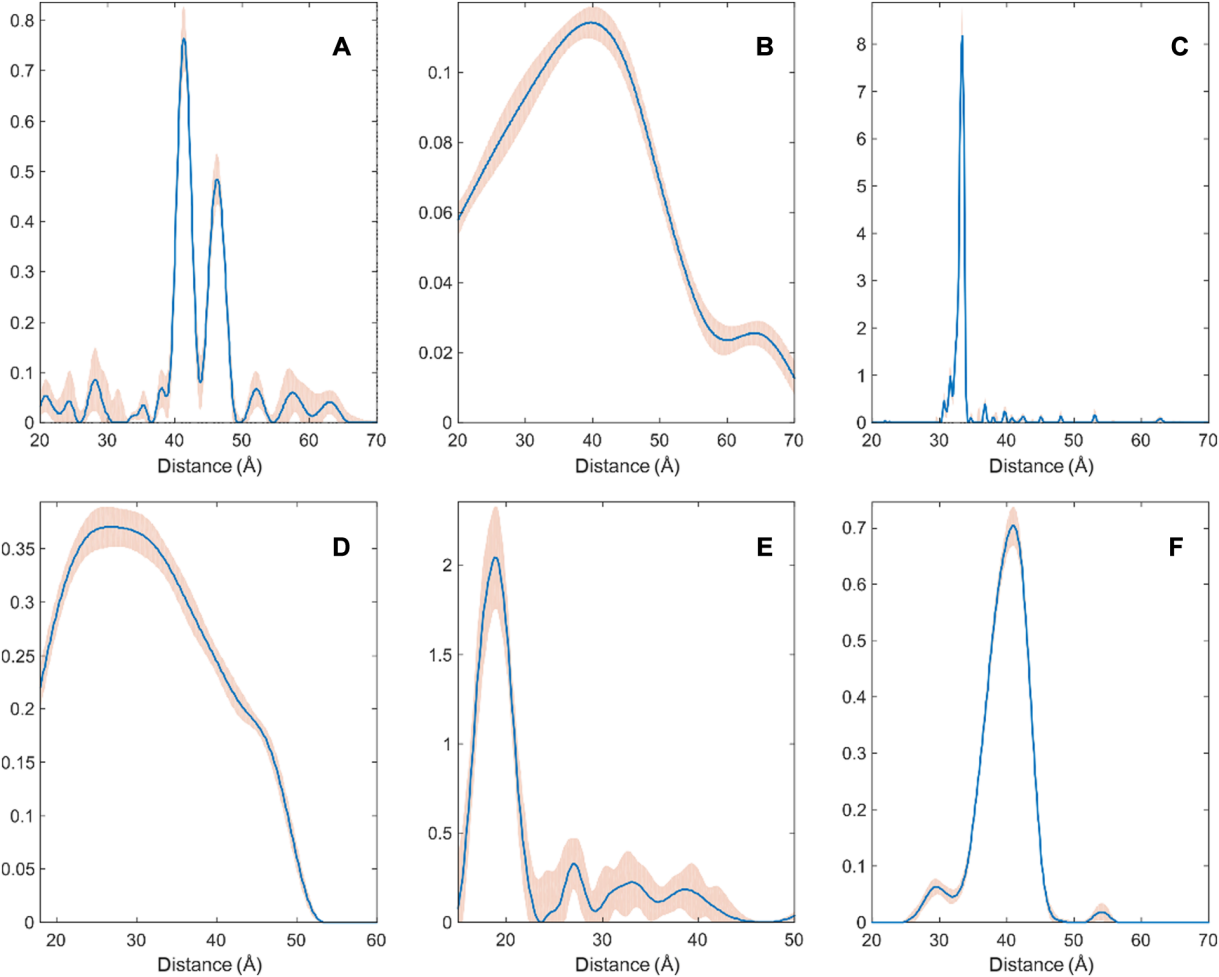
Distance distributions obtained by Tikhonov regularization (blue lines) and uncertainties estimated by the DeerAnalysis validation tool (pink areas) for the six experimental test cases. (**A**) Site pair V96C/I143C in the lumenal loop of a double mutant of LHCII, with iodoacetamido-PROXYL spin labels attached to the indicated cysteines (*64*); (**B**) site pair S3C/S34C in the N-terminal domain of a double mutant of the LHCII monomers, with iodoacetamido-PROXYL spin labels attached to the indicated cysteines (*64*); (**C**) end-labeled oligo(*para*-phenyleneethynylene)—a rigid linear molecule described as compound 3a in (*37*); (**D**) [2]catenane (a pair of large interlocked rings) with a nitroxide spin label on each ring described as sample II in (*65*); (**E**) pairs of nitroxide radicals tethered to the surface of gold nanoparticles, with the thiol tether attachment points diffusing on the surface of the nanoparticle, sample Au3 after solvolysis and heating in (*66*); (**F**) rigid molecular triangle labeled with nitroxide radicals on two corners out of three, sample B11_inv_ in (*16*).

For comparison, we also fully processed the data using DeerAnalysis (Fig. 10). We applied default background fitting, assuming a homogeneous spatial distribution (*n* = 3), except for sample III, where *n* was fitted. This exception was required because we averaged the data for sample III over 37 different observer fields to reduce orientation selection effects; this averaging causes nonexponential background decay. We found *n* = 3.40 for that sample. We then computed the L-curve in all cases. The default choice of the optimum regularization parameter (minimum distance to the origin) was accepted unless it differed clearly from the maximum curvature point and the back-predicted DEER data were clearly overdamped compared to the experimental curve. In this case, which was encountered for Sample I (see Fig. 1) and III, we selected the maximum curvature point.

We performed Monte Carlo validation by varying the noise (twice the original noise level, 11 instances) and the starting time of the background fit (from 240 ns to half the maximum time, 11 instances), giving a total of 121 Monte Carlo instances. For Sample III, we also varied the background dimension from 2.6 to 3.6 (11 instances) and reduced the number of noise instances to two per background starting time/dimension pair, giving a total of 242 instances. We pruned validation data at the default level of 1.15, meaning that all solutions with a root mean square deviation (RMSD) of the fit from the background-corrected data exceeding 1.15 times the minimum RMSD were excluded. In all cases, this pruning led to only a slight reduction of the uncertainty estimate. For Sample V, we also fitted the model of biradicals distributed on the surface of spherical particles with a Gaussian distribution of the particle radius (model Chechik2 in DeerAnalysis) (*16*). We found a biradical distance of 1.87 nm with an SD of 0.22 nm and a fraction of 0.72 for the biradical distance contribution. The particle mean radius was 4.24 nm, and its SD was 0.49 nm.

### Neural network performance

**Fig. 11:**
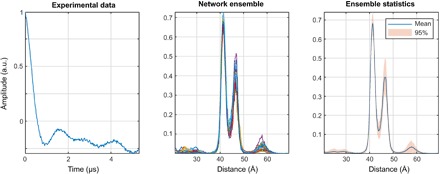
DEERNet performance on sample I: A site pair V96C/I143C in the lumenal loop of a double mutant of LHCII, with iodocateamido-PROXYL spin labels attached to the indicated cysteines (*64*). Residue 96 is located in the lumenal loop, and residue 143 is a structurally rigid “anchor” position in the protein core. In agreement with the results reported in the original paper, a bimodal distance distribution is measured—indicating flexibility in the lumenal loop. The low-confidence peak around 57 Å likely results from protein aggregation.

The DEERNet result for Sample I is shown in Fig. 11. Apart from the more generous confidence intervals reported by the neural network ensemble, there is essentially no difference from the Tikhonov result—both major distances are discovered and there is some uncertainty around the baseline. In this particular case, the performance of the two methods is identical up to the SD quoted.

**Fig. 12:**
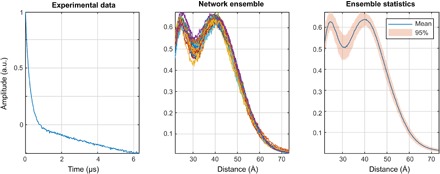
DEERNet performance on sample II: A site pair S3C/S34C in the N-terminal domain of a double mutant of the LHCII, with iodoacetamido-PROXYL spin labels attached to the indicated cysteines (*64*). The data stem from LHCIII monomers. Residue 3 is located in the very flexible N-terminal region, while residue 34 is located in the structured part of the N-terminal domain.

In Sample II, one label is situated in the structured part of the N-terminal domain of LHCII (residue 34), whereas the other one is situated near the N terminus (residue 3) in a disordered region that extends at least to residue 12. A broad distance distribution, as it was found by both Tikhonov regularization (Fig. 10B) and the neural networks (Fig. 12), is expected. A bimodal distribution produced by DEERNet cannot be excluded a priori because the “correct” answer is not known in this case.

**Fig. 13:**
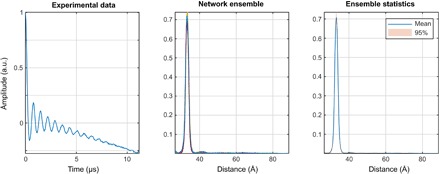
DEERNet performance on sample III: End-labeled oligo(*para*-phenyleneethynylene)—a rigid linear molecule described as compound 3a in (*37*). The maximum and the width of the distance distribution are in close agreement with the Tikhonov regularization results, whereas the expected skew of the distribution is not reproduced. Notably, there are no low-intensity artifacts that the Tikhonov method produces around the baseline.

The Tikhonov method performs better than neural networks for the very narrow and skewed distribution case seen in sample III (Fig. 13). Although skewed distributions are present in the training database, neural networks still predict a symmetric peak (at the right distance), whereas the Tikhonov output is correctly skewed, as expected for the rigid linker between the two labels that behaves as a worm-like chain (Fig. 10C). The likely reason for the loss of skew by the neural networks is insufficient point count: Our networks are only 256 neurons wide, but more points are required to reproduce the sharp features seen in Fig. 10C. Networks that are 512 or 1024 neurons wide would likely get the skew right, but training these networks would require 10 times the processing power—this will have to wait until Tesla V100 cards arrive at our local supercomputing center.

**Fig. 14:**
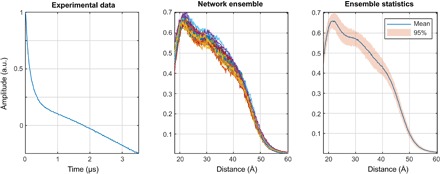
DEERNet performance on Sample IV: [2]catenane (a pair of large interlocked rings) with a nitroxide spin label on each ring. The distance distribution is in line with rough statistical estimates [Figure 5 in (*65*)], but there are fewer clumping artifacts compared to the output of the automatic Tikhonov regularization procedure. Within the Tikhonov framework, a manual regularization coefficient adjustment away from the corner of the L-curve is necessary to produce a distribution free of clumping artifacts.

Returning to broad distance distributions, the two interlocked rings in [2]catenane (Fig. 14) do perhaps push the limit of how broad a distance distribution between a pair of nitroxide radicals can be without any complications associated with exchange couplings. The original paper (*65*) reports statistical estimates of the distance distribution, but the one reported in that paper was based on the approximate Pake transformation and therefore plagued by the subjective choice of distance-domain smoothing—a fairer comparison is to the present-day Tikhonov result with the regularization parameter determined by the L-curve, as shown in Fig. 10D. Within the SDs quoted by both methods, the neural network output is not in any obvious way different from the Tikhonov regularization result. For sample IV, both approaches perform equally well within the uncertainty expected for the true distribution.

Here, some discussion is in order about the choice of the regularization parameter within the Tikhonov method. Although the L-curve criterion, on either the maximum curvature or the minimum distance to the origin, looks reassuringly algebraic, its only real justification is philosophical—a balance must be struck between the quality of fit and the regularization signal, and some humans have at some point decided that a few specific special points on the L-curve look like they strike a kind of balance. An element of human discretion is therefore always present in Tikhonov methods, as is evident from Fig. 1. Optimal choice of the regularization parameter by different approaches has recently been studied for a large set of test data, and better options than L-curve–based criteria appear to exist (*67*). On the other hand, the performance of neural networks heavily depends on the quality and the scope of the training set, which is also subject to human discretion. It would not, therefore, be fair to say that neural network results are entirely free of the human factor, but it is a human factor of a different kind.

**Fig. 15:**
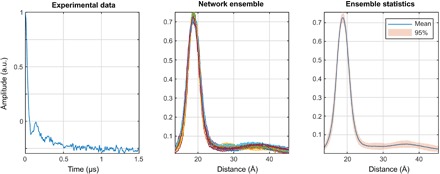
DEERNet performance on sample V: Pairs of nitroxide radicals tethered to the surface of gold nanoparticles, with the thiol tether attachment points diffusing on the surface of the nanoparticle (*66*). Note the markedly better performance relative to the Tikhonov method: The complete absence of clumping artifacts and the remarkable match to the analytical model—down to the maximum exhibited by the broad feature around 35 Å.

**Fig. 16:**
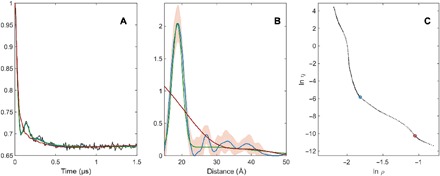
Tikhonov distance distribution analysis for pairs of nitroxide radicals tethered to the surface of gold nanoparticles, with the thiol tether attachment points diffusing on the surface of the nanoparticle [sample Au3 after solvolysis and heating in (*66*)]. Green lines correspond to a model fit assuming a Gaussian distribution of distances and a homogeneous distribution of the biradicals on spherical nanoparticles with a Gaussian distribution of radii. Blue lines correspond to Tikhonov regularization with the regularization parameter in the L-curve corner as suggested by DeerAnalysis. Red lines correspond to Tikhonov regularization with a larger regularization parameter corresponding to the second L-curve corner. (**A**) Fits of the background-corrected DEER data (black). (**B**) Distance distributions. (**C**) L-curve and the two points selected for Tikhonov distance distribution analysis.

The most impressive performance of neural networks in our test set is shown in Fig. 15—the relatively narrow peak sitting directly on top of a broad (but very real) pedestal. Tikhonov regularization has proven incapable of handling such cases [further examples may be found in (*68*)], and neither of the two corners of the L-curve (or any point anywhere else, for that matter) produces the right answer, which we know from fitting a parameterized model that agrees with known parameters of the gold nanoparticles (Fig. 16B, green curve). When a broad peak overlaps with a narrow one, the Tikhonov regularization parameter can only shift the solution between artificial broadening of the narrow peak and artificial splitting in the broad peak. Neural networks confidently produce the right answer.

Finally, for sample VI, the results of Tikhonov regularization and DEERNet agree rather nicely, except for a noise-related peak near 54 Å and a minor peak near 30 Å that appear only in the Tikhonov-derived distribution. Width and shape of the main peak are rather similar. The significance of the minor peak near 30 Å cannot be established, since molecular dynamics simulations performed for an isolated molecule at 298 K were not conclusive. Hence, the quality of the distance distributions generated by Tikhonov regularization and by the neural network should, in this case, be judged as similar.

**Fig. 17:**
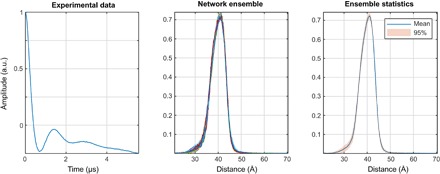
DEERNet performance on sample VI: A rigid molecular triangle labeled with nitroxide radicals on two out of three corners (*16*).

On the basis of this small but very diverse set of test cases, we can conclude that the performance of a neural network ensemble matches the performance of a software package developed over a decade. Tikhonov regularization is better at reproducing the shape of very narrow distributions and possibly also for the broadest distribution encountered, but neural networks show much better performance for distributions that feature both narrow and broad components—a case that is likely to occur in the context of order-disorder equilibria of proteins. Neural networks also appear to have an advantage in rejection of small, noise-related peaks. These features are particularly impressive when considering that the networks can be trained in a matter of hours by an unattended process. Given the close algebraic match described in Introduction, this is perhaps to be expected. Still, this begs the question of what wider and deeper networks with more sophisticated structure could accomplish. We do not, at the moment, have the computing power to explore this matter, but the “noisy” appearance of some neural network outputs in Figs. 11 to 17 suggests that further improvements are possible if the networks are trained longer and on larger data sets that are currently beyond the capacity of our Tesla cards.

### Exchange-resilient neural networks

**Fig. 18:**
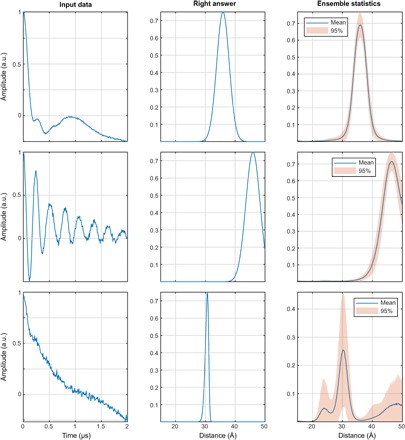
A demonstration of exchange coupling resilience. The networks were trained on the database where each DEER trace has an exchange coupling randomly selected within the ±5-MHz interval (top row, *J* = –1.9 MHz; middle row, *J* = +2.9 MHz; bottom row, *J* = –3.6 MHz) and all other parameters as described in the “Training database generation” section. More than 99% of the training data set (including distributions with multiple distance peaks) produces the results of the kind shown in the top and middle panels—fast exchange oscillations are rejected and correct distance distributions are produced. With very noisy data (bottom), the networks duly report being highly uncertain.

Neural networks successfully process cases that are completely out of design specifications of Tikhonov regularization methods—in this section, we present the results of training an ensemble of networks on data sets that include random interelectron exchange couplings selected from the user-specified range (we have used ±5.0 MHz). Typical outcomes from previously unseen synthetic data sets are shown in the top and middle rows of Fig. 18. Exchange-type distortions are prominent in the input DEER traces, but the answers produced by the networks are not perturbed.

Tikhonov regularization with a dipolar kernel returns incorrect distance distributions (fig. S7), and this failure cannot be recognized by the validation approach currently implemented in DeerAnalysis because the fit to the form factor can still appear to be good. Tikhonov methods that would account for the exchange coupling do not exist and would be exceedingly hard to create because the exchange coupling effectively adds the second dimension to the solution space.

In contrast, only the correct distances are returned by the neural networks. The rapid and slowly decaying modulation in the middle panel should have produced a short distance with a sharp peak, yet the broad peak at a large distance is correctly identified. The networks appear to learn the difference between sine/cosine and Fresnel modulations in Eq. 2, and are able to demodulate the exchange component, leaving only the dipolar part that is consistent between the sine/cosine and the Fresnel parts.

This is an impressive feat that makes DEER distance determination applicable to exchange-coupled systems that are not accessible to Tikhonov methods. Even when the networks cannot make sense of the data due to a combination of noise, exchange, and low modulation depth (Fig. 18, bottom), they still fail gracefully and report that none of the generated curve is certain. This being a clear extension of the available DEER analysis functionality, exchange-resilient neural networks will be implemented into DeerAnalysis as an option in the near future.

Including exchange resilience into the training data set costs nothing and introduces no extra work or adjustable parameters. The confidence bounds on the distance distributions coming out of exchange-resilient networks are wider, but that is to be expected because the uncertainty is increased. Another pertinent matter is that the exchange coupling can itself be distance-dependent—our current training set assumes that it is fixed. As long as the SD of the distribution is much smaller than its mean, this is a reasonable assumption.

## CONCLUSIONS AND OUTLOOK

There is a straightforward map between the algebraic structure of the two-electron dipolar spectroscopy analysis problem and the operations performed by artificial neural networks. When applied to the extraction of distance distributions from DEER traces, this produces remarkably good performance that is on par with state-of-the-art tools. We strongly recommend neural networks for cases where narrow and broad features are simultaneously present in the distance distribution. These cases can be identified by the inconclusive L-curve, such as the one in Fig. 16C. Neural networks can also return a measure of uncertainty and learn patterns of systematic distortions: A good example is the difference between an exchange coupling (pure sinusoidal pattern) and a dipolar coupling (sinusoidal + Fresnel pattern). A sufficiently deep network trained on a representative data set is able to distinguish the two and return the correct distance distribution even for exchange-coupled electrons.

At a more abstract and speculative level, the procedure described in this work effectively converts the ability to simulate a physical process into the ability to interpret experimental data. In particular, a trained neural network may be viewed as a Fredholm solver with a very general kind of regularization. Where the Tikhonov method only incorporates one of the many physical insights that humans have about the solution (namely, that it should be smooth and sparse), a perfectly trained neural network learns the entire class of admissible output patterns and only looks for solutions in that class. The challenge is rather to construct training sets that completely cover both the solution space and the distortion space that one would encounter in practice.

## Supplementary Material

http://advances.sciencemag.org/cgi/content/full/4/8/eaat5218/DC1

## SUPPLEMENTARY MATERIALS

Supplementary material for this article is available at http://advances.sciencemag.org/cgi/content/full/4/8/eaat5218/DC1 Section S1. DEER kernel derivation Section S2. Performance illustrations for networks of different depth Section S3. Effects of transfer functions, choke points, and bias vectors Section S4. Behavior of Tikhonov regularization for exchange-coupled systems Section S5. Behavior of neural networks with the increasing level of noise Fig. S1. DEERNet performance illustration, distance distribution recovery: two-layer feedforward network, fully connected, with 256 neurons per layer. Fig. S2. DEERNet performance illustration, distance distribution recovery: three-layer feedforward network, fully connected, with 256 neurons per layer. Fig. S3. DEERNet performance illustration, distance distribution recovery: four-layer feedforward network, fully connected, with 256 neurons per layer. Fig. S4. DEERNet performance illustration, form factor recovery: two-layer feedforward network, fully connected, with 256 neurons per layer. Fig. S5. DEERNet performance illustration, form factor recovery: three-layer feedforward network, fully connected, with 256 neurons per layer. Fig. S6. DEERNet performance illustration, form factor recovery: four-layer feedforward network, fully connected, with 256 neurons per layer. Fig. S7. Tikhonov analysis of synthetic data produced as described in the main text and featuring a unimodal distance distribution in the presence of a fixed exchange coupling (cf. Fig. 17). Fig. S8. A randomly generated DEER data set with the noise SD set at 2.5% of the modulation depth and the resulting distance distribution reconstruction by DEERNet. Fig. S9. A randomly generated DEER data set with the noise SD set at 10% of the modulation depth and the resulting distance distribution reconstruction by DEERNet. Fig. S10. A randomly generated DEER data set with the noise SD set at 30% of the modulation depth and the resulting distance distribution reconstruction by DEERNet. Table S1. Distance distribution recovery performance statistics for feedforward networks with hyperbolic tangent sigmoid (tansig) and logistic sigmoid (logsig) transfer function at the last layer. Table S2. Performance statistics for a family of feedforward networks set up as a sequence of fully connected layers with a choke point in the position indicated.
