# Supplementary material for: Deep neural network processing of DEER data
Source: Sci Adv. 2018 Aug 24;4(8):eaat5218. doi: 10.1126/sciadv.aat5218 (PMC6108566; doi:10.1126/sciadv.aat5218)
Supplement: http://advances.sciencemag.org/cgi/content/full/4/8/eaat5218/DC1 [file supp_4_8_eaat5218__index.html]

Science Advances | Science Advances

## Supplementary Materials

**This PDF file includes:**

- Section S1. DEER kernel derivation
- Section S2. Performance illustrations for networks of different depth
- Section S3. Effects of transfer functions, choke points, and bias vectors
- Section S4. Behavior of Tikhonov regularization for exchange-coupled systems
- Section S5. Behavior of neural networks with the increasing level of noise
- Fig. S1. DEERNet performance illustration, distance distribution recovery: two-layer feedforward network, fully connected, with 256 neurons per layer.
- Fig. S2. DEERNet performance illustration, distance distribution recovery: three-layer feedforward network, fully connected, with 256 neurons per layer.
- Fig. S3. DEERNet performance illustration, distance distribution recovery: four-layer feedforward network, fully connected, with 256 neurons per layer.
- Fig. S4. DEERNet performance illustration, form factor recovery: two-layer feedforward network, fully connected, with 256 neurons per layer.
- Fig. S5. DEERNet performance illustration, form factor recovery: three-layer feedforward network, fully connected, with 256 neurons per layer.
- Fig. S6. DEERNet performance illustration, form factor recovery: four-layer feedforward network, fully connected, with 256 neurons per layer.
- Fig. S7. Tikhonov analysis of synthetic data produced as described in the main text and featuring a unimodal distance distribution in the presence of a fixed exchange coupling (cf. Fig. 17).
- Fig. S8. A randomly generated DEER data set with the noise SD set at 2.5% of the modulation depth and the resulting distance distribution reconstruction by DEERNet.
- Fig. S9. A randomly generated DEER data set with the noise SD set at 10% of the modulation depth and the resulting distance distribution reconstruction by DEERNet.
- Fig. S10. A randomly generated DEER data set with the noise SD set at 30% of the modulation depth and the resulting distance distribution reconstruction by DEERNet.
- Table S1. Distance distribution recovery performance statistics for feedforward networks with hyperbolic tangent sigmoid (tansig) and logistic sigmoid (logsig) transfer function at the last layer.
- Table S2. Performance statistics for a family of feedforward networks set up as a sequence of fully connected layers with a choke point in the position indicated.

Download PDF

**Files in this Data Supplement:**

- Adobe PDF - aat5218\_SM.pdf
