# Supplementary material for: Deep neural network processing of DEER data
Source: Sci Adv. 2018 Aug 24;4(8):eaat5218. doi: 10.1126/sciadv.aat5218 (PMC6108566; doi:10.1126/sciadv.aat5218)
Supplement: http://advances.sciencemag.org/cgi/content/full/4/8/eaat5218/DC1 [file aat5218_SM.pdf]

Science Advances
AAAS

advances.sciencemag.org/cgi/content/full/4/8/eaat5218/DC1

Supplementary Materials for

Deep neural network processing of DEER data

Steven G. Worswick, James A. Spencer, Gunnar Jeschke, Ilya Kuprov*

*Corresponding author. Email: i.kuprov@soton.ac.uk

Published 24 August 2018, *Sci. Adv.* **4**, eaat5218 (2018)
DOI: 10.1126/sciadv.aat5218

This PDF file includes:

Section S1. DEER kernel derivation
Section S2. Performance illustrations for networks of different depth
Section S3. Effects of transfer functions, choke points, and bias vectors
Section S4. Behavior of Tikhonov regularization for exchange-coupled systems
Section S5. Behavior of neural networks with the increasing level of noise
Fig. S1. DEERNet performance illustration, distance distribution recovery: two-layer feedforward network, fully connected, with 256 neurons per layer.
Fig. S2. DEERNet performance illustration, distance distribution recovery: three-layer feedforward network, fully connected, with 256 neurons per layer.
Fig. S3. DEERNet performance illustration, distance distribution recovery: four-layer feedforward network, fully connected, with 256 neurons per layer.
Fig. S4. DEERNet performance illustration, form factor recovery: two-layer feedforward network, fully connected, with 256 neurons per layer.
Fig. S5. DEERNet performance illustration, form factor recovery: three-layer feedforward network, fully connected, with 256 neurons per layer.
Fig. S6. DEERNet performance illustration, form factor recovery: four-layer feedforward network, fully connected, with 256 neurons per layer.
Fig. S7. Tikhonov analysis of synthetic data produced as described in the main text and featuring a unimodal distance distribution in the presence of a fixed exchange coupling (cf. Fig. 17).
Fig. S8. A randomly generated DEER data set with the noise SD set at 2.5% of the modulation depth and the resulting distance distribution reconstruction by DEERNet.
Fig. S9. A randomly generated DEER data set with the noise SD set at 10% of the modulation depth and the resulting distance distribution reconstruction by DEERNet.
Fig. S10. A randomly generated DEER data set with the noise SD set at 30% of the modulation depth and the resulting distance distribution reconstruction by DEERNet.
Table S1. Distance distribution recovery performance statistics for feedforward networks with hyperbolic tangent sigmoid (tansig) and logistic sigmoid (logsig) transfer function at the last layer.

Table S2. Performance statistics for a family of feedforward networks set up as a sequence of fully connected layers with a choke point in the position indicated.

Supplementary Information

Section S1. DEER kernel derivation

A two-electron system with a dipole-dipole and an exchange coupling has the following Hamiltonian

$$\hat{H}(\Omega)=\omega_1\hat{L}_{\mathrm{Z}}^{(1)}+\omega_2\hat{L}_{\mathrm{Z}}^{(2)}-\sqrt{6}\frac{\mu_0}{4\pi}\frac{\gamma_1\gamma_2\hbar}{r^3}\sum_{m=-2}^{2}\hat{T}_{2,m}\mathfrak{D}_{m,0}^{(2)}(\Omega)+ \\ +J\left[\hat{L}_{\mathrm{X}}^{(1)}\hat{L}_{\mathrm{X}}^{(2)}+\hat{L}_{\mathrm{Y}}^{(1)}\hat{L}_{\mathrm{Y}}^{(2)}+\hat{L}_{\mathrm{Z}}^{(1)}\hat{L}_{\mathrm{Z}}^{(2)}\right] \quad (1)$$

Where Ω is a set of three Euler angles, $\mathfrak{D}_{m,0}^{(2)}(\Omega)$ are second-rank Wigner D-functions, J is defined in the NMR convention in angular frequency units, and the irreducible spherical tensor operators are

$$\hat{T}_{2,\pm2}=+\frac{1}{2}\hat{L}_{\pm}^{(1)}\hat{L}_{\pm}^{(2)},\qquad \hat{T}_{2,\pm1}=\mp\frac{1}{2}\left(\hat{L}_{\mathrm{Z}}^{(1)}\hat{L}_{\pm}^{(2)}+\hat{L}_{\pm}^{(1)}\hat{L}_{\mathrm{Z}}^{(2)}\right) \\ \hat{T}_{2,0}=+\sqrt{\frac{2}{3}}\left(\hat{L}_{\mathrm{Z}}^{(1)}\hat{L}_{\mathrm{Z}}^{(2)}-\frac{1}{4}\left(\hat{L}_{+}^{(1)}\hat{L}_{-}^{(2)}+\hat{L}_{-}^{(1)}\hat{L}_{+}^{(2)}\right)\right) \quad (2)$$

Applying the rotating frame with respect to the Zeeman Hamiltonian

$$\hat{H}_0=\omega_1\hat{L}_{\mathrm{Z}}^{(1)}+\omega_2\hat{L}_{\mathrm{Z}}^{(2)} \quad (3)$$

and relying on the fact that the spin echo would refocus the *g*-factor offset variations, we obtain the following rotating frame Hamiltonian

$$\hat{H}^{(\mathrm{R})}=\frac{\mu_0}{4\pi}\frac{\gamma_1\gamma_2\hbar}{r^3}\left[1-3\cos^2(\theta)\right]\hat{L}_{\mathrm{Z}}^{(1)}\hat{L}_{\mathrm{Z}}^{(2)}+J\hat{L}_{\mathrm{Z}}^{(1)}\hat{L}_{\mathrm{Z}}^{(2)}= \\ =D\left[1-3\cos^2(\theta)\right]\hat{L}_{\mathrm{Z}}^{(1)}\hat{L}_{\mathrm{Z}}^{(2)}+J\hat{L}_{\mathrm{Z}}^{(1)}\hat{L}_{\mathrm{Z}}^{(2)},\qquad D=\frac{\mu_0}{4\pi}\frac{\gamma_1\gamma_2\hbar}{r^3} \quad (4)$$

Here we have skipped all but the secular components, which is permitted when $|\omega_1-\omega_2|\gg|J|,D$. This condition is fulfilled in DEER for most spin pairs if the difference between the pump and observe frequencies strongly exceeds *J* and *D*. The Hamiltonian given in Eq. (4), when acting on the transverse magnetization, produces the following oscillation

$$s(\theta,t)=\cos\left[\left(D\left[1-3\cos^2(\theta)\right]+J\right)t\right] \quad (5)$$

Averaging this over all molecular orientation produces the required formula

$$\gamma(D,J,t)=\int_0^{\pi}\cos\left[\left(D\left[1-3\cos^2(\theta)\right]+J\right)t\right]\sin\theta d\theta=$$
$$=\sqrt{\frac{\pi}{6Dt}}\left[\cos\left[(D+J)t\right]\mathrm{FrC}\left[\sqrt{\frac{6Dt}{\pi}}\right]+\sin\left[(D+J)t\right]\mathrm{FrS}\left[\sqrt{\frac{6Dt}{\pi}}\right]\right] \tag{6}$$

where Fresnel functions are defined as

$$\mathrm{FrC}(x)=\int_0^x\cos\left(t^2\right)dt \qquad \mathrm{FrS}(x)=\int_0^x\sin\left(t^2\right)dt \tag{7}$$

Equation (6) still has a simple analytical form when a Gaussian distribution in the exchange coupling is assumed, because the exchange coupling only occurs under simple trigonometric functions, for which

$$\frac{1}{\sqrt{2\pi}\sigma_J}\int_{-\infty}^{\infty}e^{-\frac{(J-J_0)^2}{2\sigma_J^2}}\cos\left[(D+J)t\right]dJ=e^{-\frac{t^2\sigma_J^2}{2}}\cos\left[(D+J_0)t\right]$$
$$\frac{1}{\sqrt{2\pi}\sigma}\int_{-\infty}^{\infty}e^{-\frac{(J-J_0)^2}{2\sigma_J^2}}\sin\left[(D+J)t\right]dJ=e^{-\frac{t^2\sigma_J^2}{2}}\sin\left[(D+J_0)t\right] \tag{8}$$

Thus, the distribution in the exchange coupling creates an extra decay in the DEER trace, but the sinusoidal modulation still occurs at the frequency $D+J_0$. The updated expression for the form factor is

$$\gamma(D,J_0,\sigma_J,t)=\sqrt{\frac{\pi}{6Dt}}\left(\cos\left[(D+J_0)t\right]\mathrm{FrC}\left[\sqrt{\frac{6Dt}{\pi}}\right]+\sin\left[(D+J_0)t\right]\mathrm{FrS}\left[\sqrt{\frac{6Dt}{\pi}}\right]\right)e^{-\frac{t^2\sigma_J^2}{2}} \tag{9}$$

where J_0 is the average exchange coupling and σ_J is the standard deviation of its distribution.

Section S2. Performance illustrations for networks of different depth

The “relative error” metric is defined as the 2-norm of the difference between the network output and the ground truth, divided by the 2-norm of the ground truth. The data for the performance illustrations were selected from the training database in the following way: the “easy case” was sampled from the region between zero and one standard deviation on the relative error; the “tough case was sampled

from the region between one and two standard deviations; the “bad case” was sampled from 100 worst fits in the entire 100,000-trace training database.

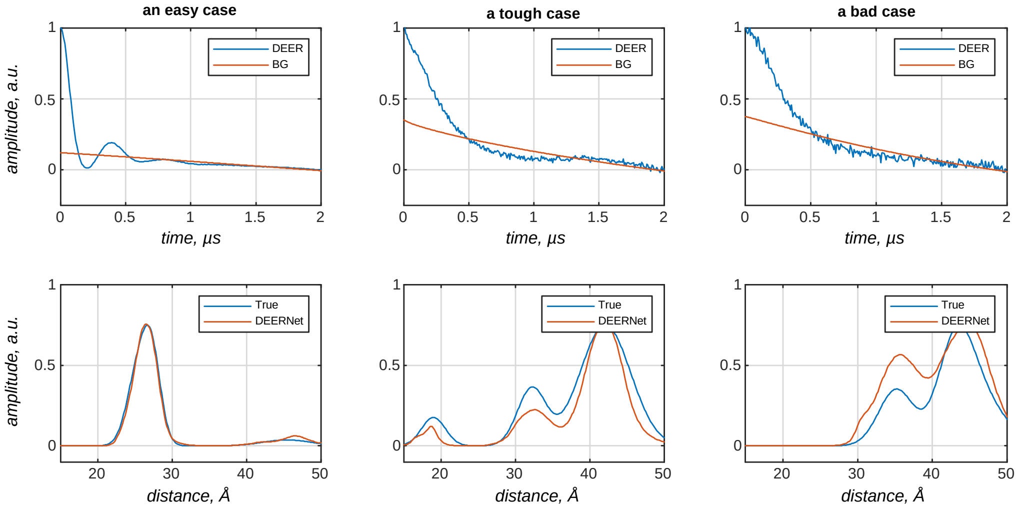

Fig. S1. DEERNet performance illustration, distance distribution recovery: two-layer feedforward network, fully connected, with 256 neurons per layer. The first layer has a sigmoid transfer function; the second layer has a log-sigmoid transfer function. BG = background.

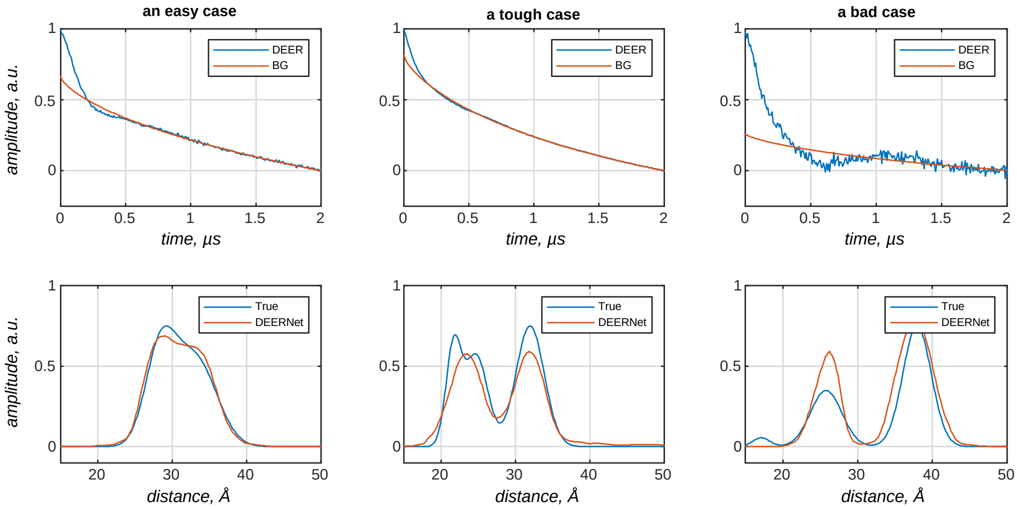

Fig. S2. DEERNet performance illustration, distance distribution recovery: three-layer feedforward network, fully connected, with 256 neurons per layer. The first two layers have a sigmoid transfer function; the last layer has a log-sigmoid transfer function. BG = background.

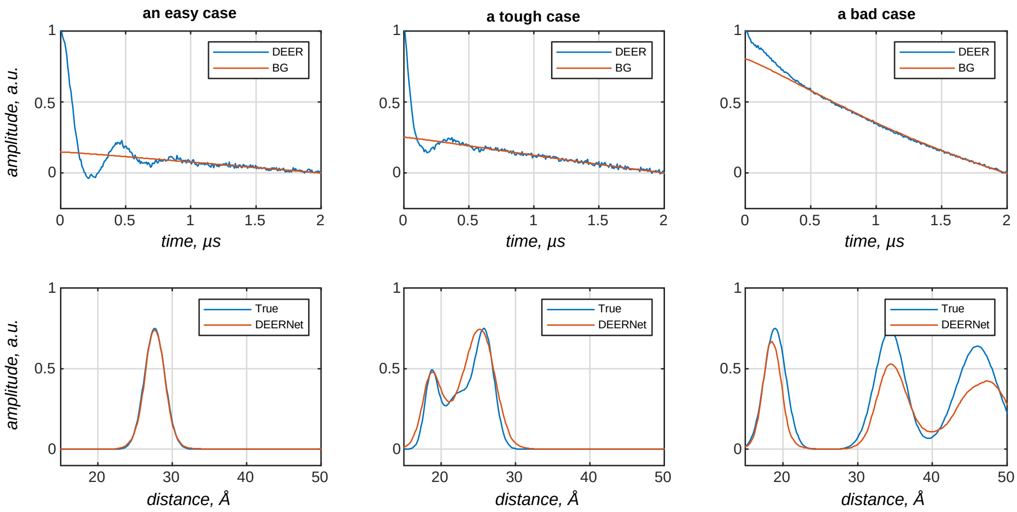

Fig. S3. DEERNet performance illustration, distance distribution recovery: four-layer feedforward network, fully connected, with 256 neurons per layer. The first three layers have a sigmoid transfer function, the last layer has a log-sigmoid transfer function. BG = background.

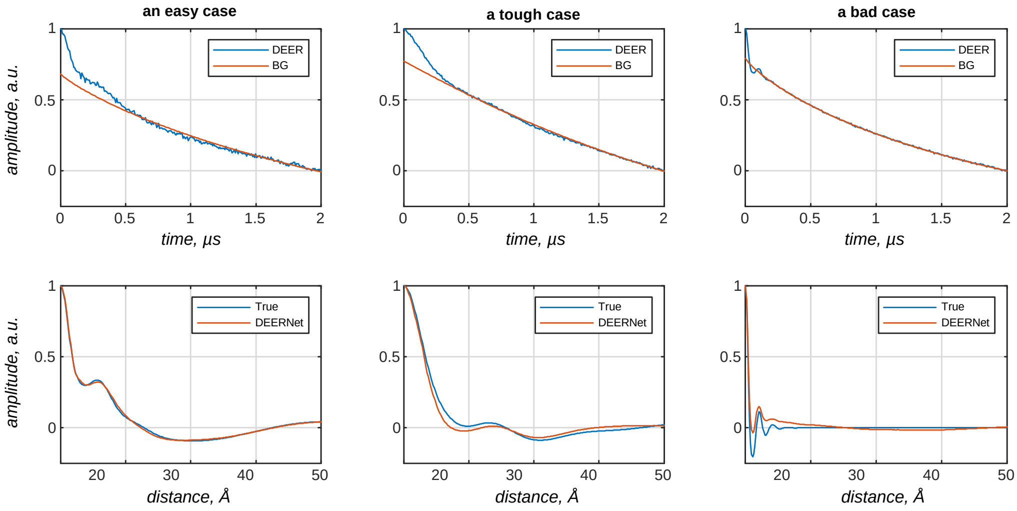

Fig. S4. DEERNet performance illustration, form factor recovery: two-layer feedforward network, fully connected, with 256 neurons per layer. All layers have a sigmoid transfer function. BG = background.

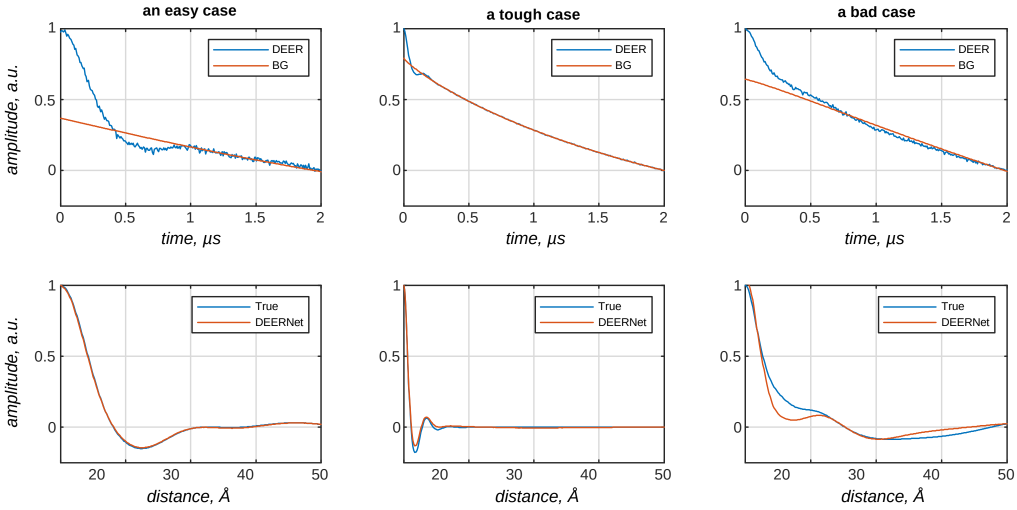

Fig. S5. DEERNet performance illustration, form factor recovery: three-layer feedforward network, fully connected, with 256 neurons per layer. All layers have a sigmoid transfer function. BG = background.

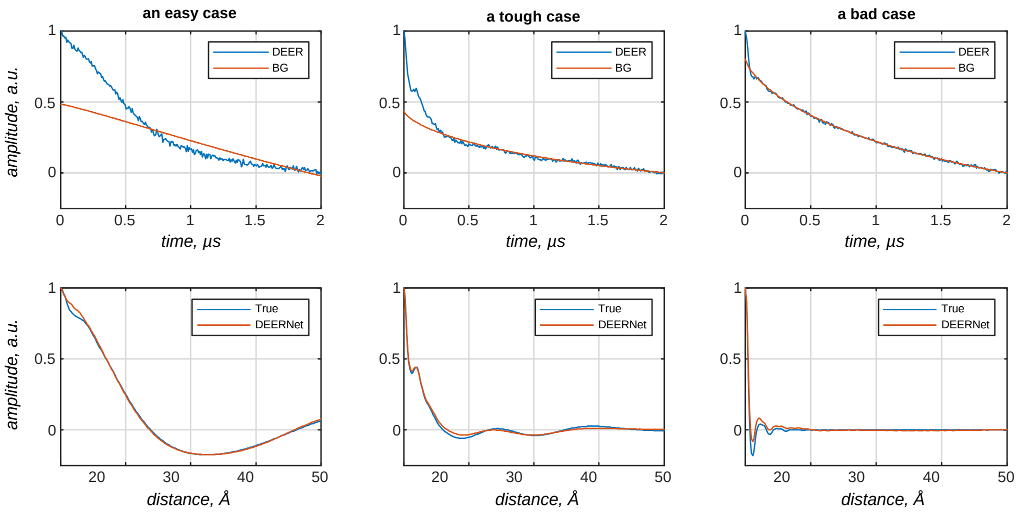

Fig. S6. DEERNet performance illustration, form factor recovery: four-layer feedforward network, fully connected, with 256 neurons per layer. All layers have a sigmoid transfer function. BG = background.

Section S3. Effects of transfer functions, choke points, and bias vectors

A minor complication associated with logistic sigmoid transfer functions – that negative inputs are mapped into very small numbers and the vanishing gradient problem becomes more severe – does not appear to be a problem for the relatively shallow networks used in this work.

Table S1. Distance distribution recovery performance statistics for feedforward networks with hyperbolic tangent sigmoid (tansig) and logistic sigmoid (logsig) transfer function at the last layer.

Network	Inner layer transfer function	Last layer transfer function	Mean relative error	Iteration count at convergence
In-$(256)_2$-Out	tansig	tansig	0.110	2915
In-$(256)_3$-Out	tansig	tansig	0.091	3134
In-$(256)_4$-Out	tansig	tansig	0.088	4570
In-$(256)_2$-Out	tansig	logsig	0.090	1062
In-$(256)_3$-Out	tansig	logsig	0.077	2234
In-$(256)_4$-Out	tansig	logsig	0.070	2495

Table S2. Performance statistics for a family of feedforward networks set up as a sequence of fully connected layers with a choke point in the position indicated. A schematic of the network topology is given in the middle diagram of Fig. 3.

Network	Bias vectors present		Bias vectors absent	
	Mean relative error	Rel. error st. dev.	Mean relative error	Rel. error st. dev.
In-256-***128***-256-256-256-256-Out	0.071	0.200	0.073	0.202
In-256-256-***128***-256-256-256-Out	0.074	0.193	0.074	0.200
In-256-256-256-***128***-256-256-Out	0.071	0.195	0.072	0.196
In-256-256-256-256-***128***-256-Out	0.072	0.194	0.072	0.198

The most likely reason for the bias vectors apparently not having any influence on the network performance is the fact that the networks in question are fully connected and the input data are well scaled. It is easy to demonstrate that the operation involving a matrix product and a bias vector:

$$\mathbf{Wx}+\mathbf{b} \qquad (10)$$

is equivalent to just a matrix-vector product involving a matrix with one extra row and column:

$$\begin{bmatrix} 1 \\ \mathbf{Wx}+\mathbf{b} \end{bmatrix} = \begin{bmatrix} 1 & \mathbf{0} \\ \mathbf{b} & \mathbf{W} \end{bmatrix} \begin{bmatrix} 1 \\ \mathbf{x} \end{bmatrix} \tag{11}$$

For a network with 256 or more neurons per layer, the accuracy impact of dedicating one line of neurons to effectively handling the bias appears to be negligible.

Section S4. Behavior of Tikhonov regularization for exchange-coupled systems

All current Tikhonov regularisation methods are based on the pure dipole-dipole coupling model; they are not expected to produce correct answers for exchange-coupled systems. Here we briefly demonstrate that this is indeed the case. We have processed the simulated data shown in Fig. 17 of the main text by Tikhonov regularization. In cases where the fit of the form factor looked reasonable, we have performed validation in *DeerAnalysis*. The results are shown in fig. S7.

For the case shown in the top row, an expert would recognize that the fit (red line in the left panel) of the form factor (black line) is too poor for the distance distribution (blue line in the right panel) to be trusted. Reckless use of a dipole-only model would produce an apparently trustworthy peak around 24 Å. This is, of course, incorrect: the underlying mean distance is about 36 Å as seen in Fig. 17 in the main text.

For the data set shown in the middle row of Fig. 17, Tikhonov regularization does not find a solution that fits the data, but the failure is graceful – it is readily apparent to a non-expert user.

For the data set shown in the bottom row of Fig. 17, Tikhonov regularization provides a fit of the form factor within the amplitude of the noise. Nothing looks suspicious, not even the *L*-curve (data not shown). The apparent distance distribution is bimodal with peaks at about 23 and 41 Å, whereas the input distribution was a narrow unimodal skewed distribution with a mean value slightly above 20 Å. In other words, the failure is not apparent, even after validation.

In applications of site-directed spin labelling to structural biology, no exchange coupling is expected in the distance range above 15 Å. However, the same does not apply in materials science where the molecular backbone may be conjugated, or the matrix conducting or semiconducting. In those cases, *DEERNet* either provides reliable data or fails gracefully, whereas analysis by Tikhonov regularization based on a purely dipolar kernel leads to incorrect results. Importantly, we have only tested fixed exchange couplings so far – data sets with a distribution over exchange couplings are likely to be much harder to process.

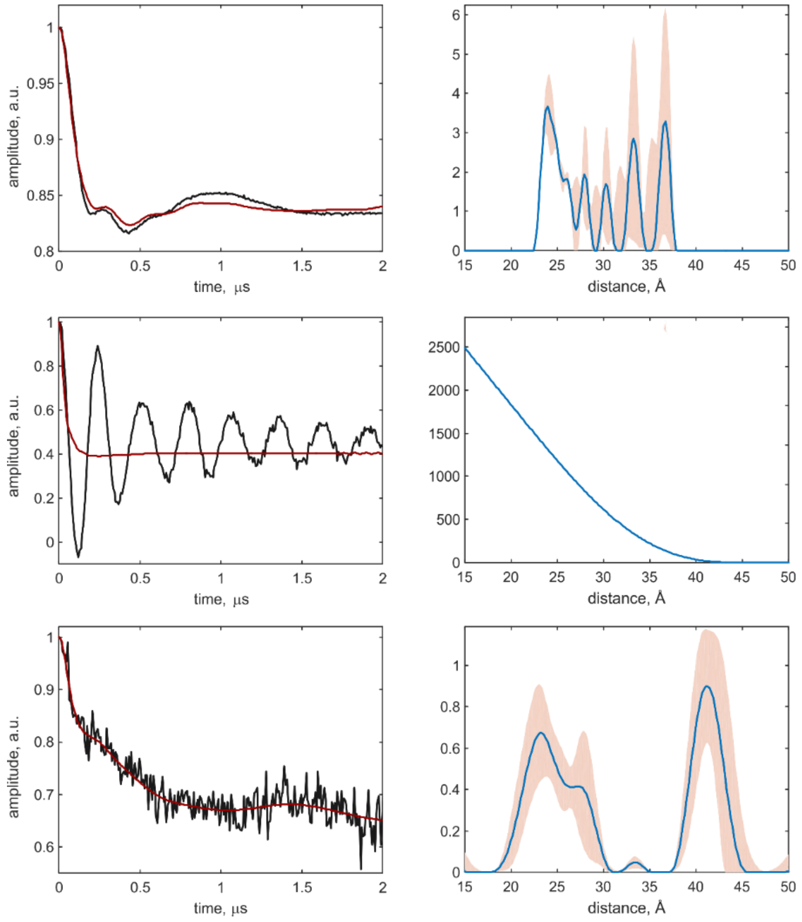

Fig. S7. Tikhonov analysis of synthetic data produced as described in the main text and featuring a unimodal distance distribution in the presence of a fixed exchange coupling (cf. Fig. 17). The left column shows the background-corrected synthetic data (black) and their fit based on the distance distribution obtained by Tikhonov regularization. The right column shows the distance distribution (blue) and, in cases where the form factor fit is reasonable, the uncertainty estimate by the validation tool of DeerAnalysis (pink shaded area).

Section S5. Behavior of neural networks with the increasing level of noise

Increasing the level of noise in the input data results in a gradual degradation in the accuracy of the distance distribution reconstruction, but also produces appropriately increased uncertainty bounds. As Figures S8-S10 illustrate, the user is not misled at any point – in all cases, the uncertainty bounds are a suitable reflection of the deviation of the reconstruction from the true answer.

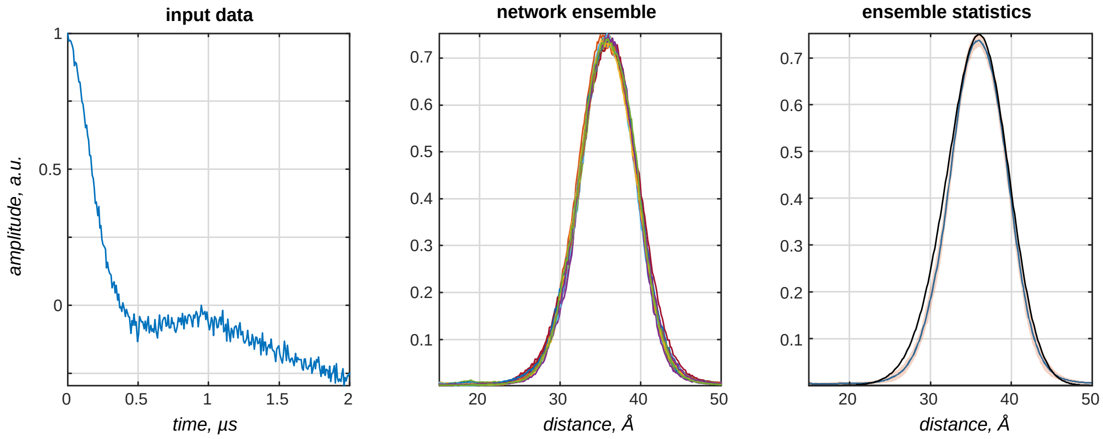

Fig. S8. A randomly generated DEER data set with the noise SD set at 2.5% of the modulation depth and the resulting distance distribution reconstruction by DEERNet. In the rightmost diagram, the blue line is the average neural network output, the shaded area is the 95% confidence interval, and the black line is the true answer.

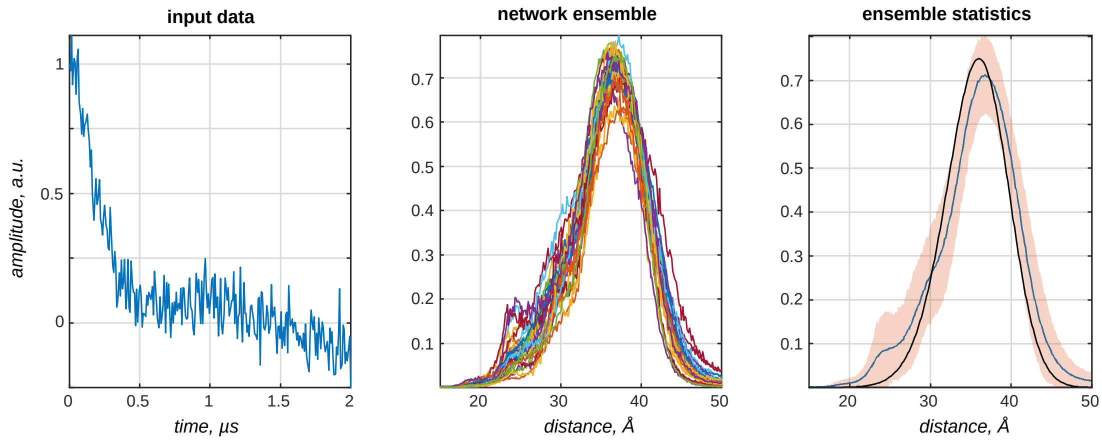

Fig. S9. A randomly generated DEER data set with the noise SD set at 10% of the modulation depth and the resulting distance distribution reconstruction by DEERNet. In the rightmost diagram, the blue line is the average neural network output, the shaded area is the 95% confidence interval, and the black line is the true answer.

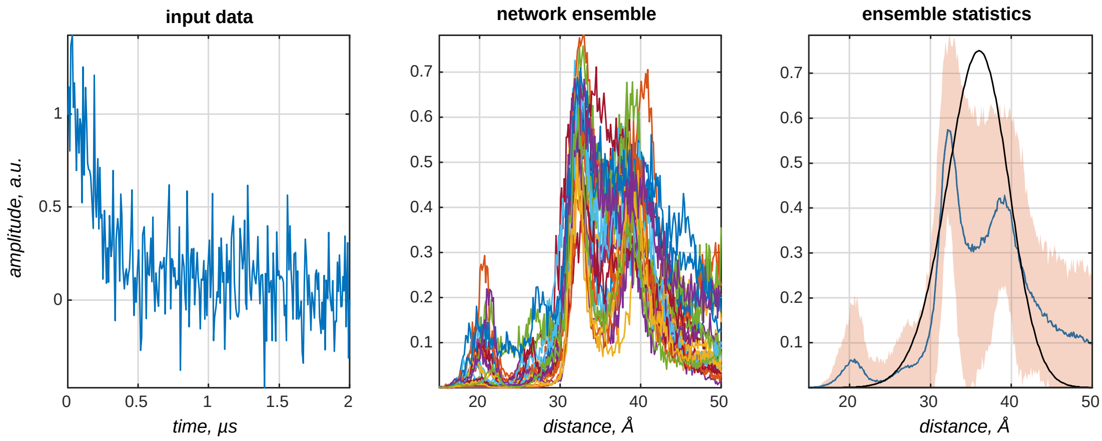

Fig. S10. A randomly generated DEER data set with the noise SD set at 30% of the modulation depth and the resulting distance distribution reconstruction by DEERNet. In the rightmost diagram, the blue line is the average neural network output, the shaded area is the 95% confidence interval, and the black line is the true answer.
